# Spirothiazolidine-Derivative on Silver Nanoparticles and Carbon Nanotubes: Evaluation of Antibacterial, Anti-Fungal, Anti-Inflammatory, Antioxidant and Gastroprotective Activities

**DOI:** 10.3390/pharmaceutics16070901

**Published:** 2024-07-05

**Authors:** Walaa I. El-Sofany, Eid. M. S. Azzam, Salman Latif, Khaled Hamden

**Affiliations:** 1 Department of Chemistry, College of Science, University of Ha’il, Ha’il 81451, Saudi Arabia; 2 Photochemistry Department, Chemical Industries Research Institute, National Research Centre, 33 EL Buhouth St., Dokki, Giza 12622, Egypt; 3 Egyptian Petroleum Research Institute, Nasr City, Cairo 11727, Egypt; 4 Laboratory of Bioresources: Integrative Biology and Exploiting, Higher Institute of Biotechnology of Monastir, University of Monastir, Monastir 5000, Tunisia

**Keywords:** spirothiazolidine, Pyridinespirothiazolidine, carbon nanotubes, microbe, inflammation, ulcer

## Abstract

This study aims to develop innovative heterocyclic nanocomposites incorporating silver nanoparticles (SNPs) for potential therapeutic applications targeting infections, gastric ulceration, inflammation, and oxidative damage. By synthesizing new derivatives of spiro-thiazolidine-carbonitrile (Py-ST-X) and incorporating them into Ag nanoparticles (AgNPs) and carbon nanotubes (CNTs), we have prepared Ag@Py-ST-X and Ag@Py-ST-X@CNT nanocomposites, respectively. The physical properties of these materials were studied using XRD, TEM, SEM, and Zeta potential techniques. In our investigation involving rats with gastric ulcers, we observed noteworthy inhibitory effects on gastric acid enzyme activity, specifically H^+^/K^+^ATPase, by Ag@Py-ST-NO_2_ and Ag@Py-ST-Br nanocomposites, demonstrating reductions of 25 and 34%, respectively, compared to untreated ulcers. Nanotubulation of these compounds further improved their inhibitory efficacy to 29 and 45%, respectively. Additionally, these nanoparticles showed the most potent myeloperoxidase (MPO)-inhibitory activity, demonstrating 36 and 49% inhibition, respectively, with nanotubulated versions reaching 44 and 53%. Moreover, Ag@Py-ST-NO_2_@CNT and Ag@Py-ST-Br@CNT nanotubes showed significant antioxidant activity, reducing thiobarbituric acid reactive substances (TBARS) by 35 and 51%, and hydrogen peroxide (H_2_O_2_) levels by 49 and 71%, respectively. These therapeutic effects were confirmed by reductions in gastric surface area (GSA) by 44% and 52%, a decrease in ulcer index (UI) from 80% to 44 and 38%, and an increase in curative index (CI) from 19 to 55 and 62% following administration of Ag@Py-ST-NO_2_@CNT and Ag@Py-ST-Br@CNT, respectively. Histological studies support these findings, suggesting the potential of these nanocomposites as promising candidates for treating various disorders.

## 1. Introduction

The primary goal of medicinal chemistry is to create, produce, and develop compounds that positively affect human health while minimizing potential toxicity [1,2,3]. Recent advancements in combinatorial chemistry have made it possible to obtain chemical libraries based on desired structures [1,2]. Nanotechnology has found applications in various sectors such as energy, the automotive and aerospace industries, textiles, agriculture, medicine, pharmaceutics, and chemistry [3,4,5,6,7]. In the field of medicine and pharmaceuticals, nanotechnology is particularly prominent, accounting for approximately 30% of all nanotechnology applications [8]. Consequently, there is a continuous and challenging pursuit to develop innovative methodologies for synthesizing carbon and silver-containing heterocycles [9]. Among these heterocyclic structures, spiro-thiazolidines are a unique class, offering a wide range of pharmaceutical properties [8]. These properties include anti-histaminic, anti-proliferative, anesthetic, hypnotic, anti-fungal, anti-inflammatory, anti-HIV, anthelmintic, CNS stimulant, and antiviral activities [8]. ST derivatives have garnered significant attention due to their exceptional properties, such as a high aspect ratio, ultra-lightweight design, high tensile strength, excellent electrical conductivity, chemical and thermal stability, and their potential to give rise to new material classes [10,11,12]. 

Carbon nanotubes (CNTs) are fullerene nanomaterials and allotropes of carbon, known for their high aspect ratios, with diameters ranging from 1 to 100 nm and lengths up to several centimeters [13]. This process results in the formation of a hybrid nanostructure that inherits characteristics from both parent structures. The fusion of nanoparticles into the tubular structure of CNTs is a highly intriguing process due to its potential implications. The unique qualities of nanocomposites have captured the interest of numerous scientists, driving further exploration and research in this field [13,14,15]. Three main aspects contributed to these: tiny size, large surface area, and quantum dimension, which can enhance their physical and chemical properties [15,16,17]. Over the last few decades, extensive research has led to the development of adaptable techniques for altering carbon nanotubes (CNTs) and producing counterparts with more desirable properties [18,19]. The remarkable chemical activity of metal nanoparticle-enhanced carbon nanotubes (CNTs) stems from their large dynamic surface area and crystallographic surface structure. These properties have been reviewed for potential uses in heterogeneous catalysis, nanoelectronics, chemical and biochemical sensors [20,21]. Physiologically, nanotubes have demonstrated a capacity to deliver bioactive components, increasing their stability and availability at the gastric level, which consequently enhances the therapeutic effect of these active components [22]. Beyond their role in drug delivery, nanotubes have potential applications in cancer treatment due to their ability to inhibit angiogenesis, thereby blocking the division of carcinogenic cells [23]. Furthermore, studies have shown that CNTs can interact with DNA, induce immune system signaling, and promote antibody production. Additionally, CNTs serve as carriers and inducers of antimicrobial agents, such as the antifungal amphotericin B, and have been shown to reduce the toxicity of antibiotics by 40% compared to the free drug [13].

The AgNP-decorated carbon nanotube (AgNP/CNT) assembly has gained significant attention in various fields due to its potential applications in antimicrobial agents, gas sensors, and electrical conductivity improvement. This new hybrid material has recently shown promise in these areas [24,25]. Metal nanoparticles on carbon nanotubes exhibit distinct electronic, optical, and catalytic properties compared to their bulk counterparts, owing to their small size. By manipulating the size, shape, and distribution of these nanoparticles, their intrinsic properties can be enhanced or modified [26]. Several methods have been established to produce composites of CNTs and silver nanoparticles, including solid-state reactions, thermal evaporation, surface chemical reduction, and wet chemical routes [27,28,29]. These techniques enable the functionalization of NPs and CNTs in a non-covalent or covalent manner. By functionalizing the sidewalls of carbon nanotubes, their potential as sensitive fabrics for detecting extremely low concentrations of biological or chemical molecules in sensing applications can be further enhanced. Moreover, this functionalization can improve the interaction between specific chemical species and the nanotubes, as well as the selectivity of the adsorption process [30]. 

Stomach ulcers, which affect approximately 10% of the global population, pose a significant challenge to global health [31]. The development of gastric ulcers involves damage to the mucosal lining primarily caused by an excess of stomach acid [28,29]. The gastric proton pump, known as the H^+^/K^+^-ATPase enzyme, plays a crucial role in the secretion of stomach acid. One approach to addressing stomach hyperacidity is to inhibit this enzyme, thereby reducing the production of gastric juice [32,33]. The gastric proton pump, or H^+^/K^+^-ATPase enzyme, is pivotal in stomach acid secretion. One strategy to address stomach hyperacidity involves inhibiting this enzyme, thereby reducing gastric juice production [34]. Inhibiting the gastric H^+^/K^+^ ATPase enzyme has proven effective in managing stomach contents, promoting healing, and alleviating symptoms in individuals with gastroesophageal reflux disease and peptic ulcers [35]. Proton pump inhibitors (PPIs), such as omeprazole, esomeprazole, and rabeprazole, are commonly prescribed to suppress acid secretion. These medications have demonstrated efficacy in treating acid-related diseases triggered by factors like aspirin, alcohol, and other substances causing hyperacidity. However, it is important to note that PPIs come with potential adverse effects, including nausea, headaches, vitamin B12 deficiency, abdominal pain, constipation, diarrhea, and osteoporosis [36,37].

Based on the aforementioned information, our primary objective is to assess the antibacterial, antifungal, and anti-inflammatory properties, as well as the inhibitory effect against gastric ulceration, of spiro(cyclohexane-thiazolidine) derivatives and related nanocomposites. This comprehensive evaluation aims to develop a physiologically relevant scaffold with potential therapeutic applications.

## 2. Materials and Methods

### 2.1. Preparation of Materials

#### 2.1.1. Chemicals

We obtained silver nitrate (AgNO_3_, 99%), *p-*bromoaniline, thioglycolic acid, *p-*chlorobenzaldehyde, *p-*nitrobenzaldehyde, or *p-*bromobenzaldehyde from Sigma-Aldrich (Munich, Germany). Additionally, 4-methylcyclohexanone (C_7_H_12_O, 99%) was purchased. All further analytical-grade chemicals were used as supplied without additional purification. Aqueous solutions were prepared using the Milli-Q3 system (Millipore, Burlington, MA, USA) with Milli-Q water (>18.2 MΩ·cm).

#### 2.1.2. Preparation of Spiro-Thiazolidine (**ST**)

**Scheme 1:**
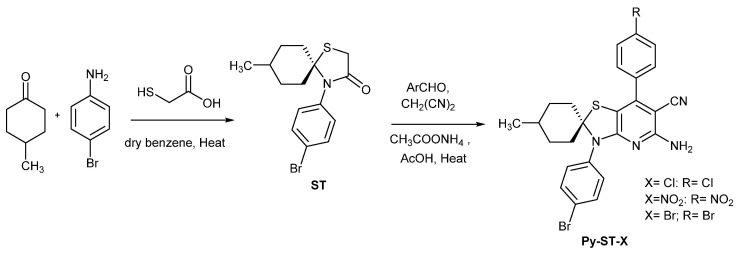
Synthesis of **Py-ST-X** derivatives.

Spiro-thiazolidine (**ST**) was synthetized according to our previous method [36,37] (Scheme 1). A solution of 4-methylcyclohexanone (0.01 mol), *p-*bromoaniline (0.01 mol), and thioglycolic acid (0.01 mol) in 50 mL of dry benzene was heated under reflux for 10 h. Compound **ST** was obtained as pale yellow needles by concentrating the reaction mixture and filtering out the produced solid, drying it, and crystallizing it from the dioxane/methanol (1:1) solution [16,38].

#### 2.1.3. Preparation of Pyridinespirothiazolidines (**Py-ST-X**)

The **Py-ST-X** derivatives were efficiently synthesized through a one-pot three-component reaction of compound **ST**, malononitrile, and various *para* substituted benzaldehydes in the presence of ammonium acetate (Scheme 1), according to a previously published procedure [39,40].

A solution of compound **ST** (0.01 mol), *p-*chlorobenzaldehyde, *p-*nitrobenzaldehyde, or *p-*bromobenzaldehyde (0.01 mol), ammonium acetate (0.02 mol), and malononitrile (0.01 mol) in glacial acetic acid (40 mL) was heated under reflux for 24 h. After the reaction mixture was cooled, it was carefully poured into water. The resulting deep yellow powder, **Py-ST-X** (X = Cl, Br, NO_2_), was obtained by filtering, drying, and crystallizing the produced solid from dioxane.

5′-amino-3′-(4-bromophenyl)-7′-(4-chlorophenyl)-4-methyl-3′H-spiro [cyclohexane-1,2′-thiazolo[4,5-b]pyridine]-6′-carbonitrile (**Py-ST-Cl**)

MP 180–182 °C, yellow powder (65%), IR (KBr, υ, cm^−1^): 3136 (NH_2_), 2221 (CN). ^1^H NMR (DMSO-*d_6_*, 300 MHz): δ (ppm) 1.47–1.9 (m, 10H, 5CH_2_), 2.46 (s, 3H, CH3), 7.10–7.69 (m, 8H, Ar–H), 8.52 (s, 2H, NH_2_, D_2_O exchangeable). ^13^C NMR (DMSO-*d_6_*, 75 MHz): δ (ppm) 22.23 (CH_3_), 30.85–40.48 (5CH_2_), 73.77 (C-spiro), 122.29 (CN), 130.26–136.46 (aromatic carbons), 160.68 (C-NH_2_). MS, m/z (%): 525.89 (M^+^, 70).

5′-amino-3′-(4-bromophenyl)-4-methyl-7′-(4-nitrophenyl)-3′H-spiro [cyclohexane-1,2′-thiazolo[4,5-b]pyridine]-6′-carbonitrile (**Py-ST-NO_2_**)

MP 205–207 °C, pale brwon powder (63%), IR (KBr, υ, cm^−1^): 3120 (NH_2_), 2216 (CN). ^1^H NMR (DMSO-*d_6_*, 300 MHz): δ (ppm) 1.48–1.97 (m, 10H, 5CH_2_), 2.35 (s, 3H, CH_3_), 7.14–7.65 (m, 8H, Ar–H), 8.56 (s, 2H, NH_2_, D_2_O exchangeable). ^13^C NMR (DMSO-*d_6_*, 75 MHz): δ (ppm) 21.34 (CH_3_), 29.31–39.48 (5CH_2_), 73.71 (C-spiro), 121.23 (CN), 131.35–135.41 (aromatic carbons), 161.61 (C–NH_2_). MS, *m*/*z* (%): 536.45 (M^+^, 73). 

5′-amino-3′,7′-bis(4-bromophenyl)-4-methyl-3′H-spiro[cyclohexane-1,2′-thiazolo[4,5-b]pyridine]-6′-carbonitrile (**Py-ST-Br**).

MP 251–253 °C, deep yellow powder (65%), IR (KBr, υ, cm^−1^): 3131 (NH_2_), 2219 (CN). ^1^H-NMR (DMSO-*d_6_*, 300 MHz): δ (ppm) 1.49–1.96 (m, 10H, 5CH_2_), 2.33 (s, 3H, CH_3_), 7.16–7.68 (m, 8H, Ar–H), 8.53 (s, 2H, NH_2_, D_2_O exchangeable). ^13^C NMR (DMSO-*d_6_*, 75 MHz): δ (ppm) 21.91 (CH_3_), 28.33–40.31 (5CH_2_), 73.25 (C-spiro), 121.82 (CN), 130.82–134.62 (aromatic carbons), 160.75 (C–NH_2_). MS, *m*/*z* (%): 570.35 (M^+^, 78).

#### 2.1.4. Preparation of AgNPs

In our previous study [39], we utilized a chemical reduction method to prepare a colloidal solution of AgNPs. The process involved boiling 0.01 g of AgNO_3_ in 50 mL of distilled water until it reached a temperature of 100 °C. Subsequently, 5 mL of a 0.01 g sodium citrate solution was added, and the mixture was further heated to ensure thorough mixing and to achieve a pale-yellow color. Once the heat was turned off, the mixture was allowed to cool down to room temperature.

#### 2.1.5. Preparation of **Ag@Py-ST-X** Composites

**Scheme 2:**
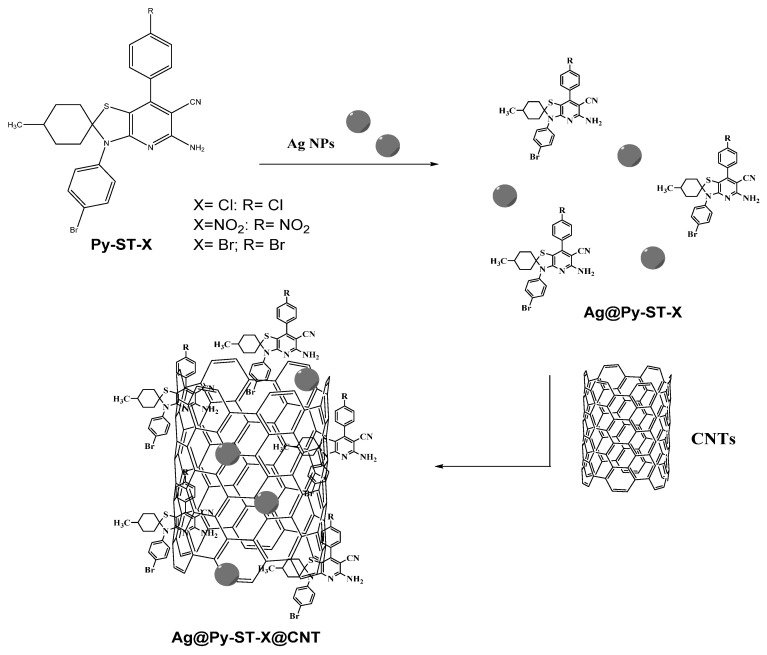
Synthesis of Ag@Py-ST-X and Ag@Py-ST-X@CNT composites.

The nanostructure of compounds **Py-ST-X** (X = Cl, Br, and NO_2_) with Ag nanoparticles (Scheme 2) was obtained using the following procedure: **Py-ST-X** compounds were dissolved in a propanol solution and stirred continuously for one hour to dissolve. Afterwards, 5 mL of the **Py-ST-X** solution was mixed with 20 mL of the Ag nanoparticles’ aqueous solution. The mixture was then stirred until the light yellow color of the AgNPs faded, result of the formation of **Ag@Py-ST-X** compounds [39].

#### 2.1.6. Preparation of **Ag@Py-ST-X@CNT** Composites

Furthermore, we prepared the **Ag@Py-ST-X@CNT** nanocomposites [38]. This involved adding the ethanolic CNT solution (1 g/100 mL) to 50 mL of Py-ST-X solution (X = Cl, Br, or NO_2_) prepared in the previous step (2.1.5). The mixture was stirred for 24 h, and the resulting material was cleaned with water and filtered. Finally, the pure nanocomposites **Ag@Py-ST-X@CNT** were obtained by drying them under vacuum overnight.

### 2.2. Material Characterization

The IR spectra were recorded using a Perkin-Elmer 1600 FTIR spectrometer (Perkin-Elmer, Waltham, MA, USA). NMR spectra were obtained at the National Research Center in Cairo, Egypt, utilizing a Jeol-Ex-300 NMR spectrophotometer (JEOL, Tokyo, Japan). Chemical shifts are reported in parts per million (ppm) relative to tetramethylsilane, which serves as an internal reference. For ^1^H and ^13^C, chemical shifts were compared to the solvent signal (dimethylsulfoxide, or DMSO) at δ 2.50 and 39.52 parts per million, respectively. The data provided include chemical shift, integration, multiplicity (br = broad, s = singlet, d = doublet, t = triplet, q = quartet, and m = multiplet), and the coupling constant in hertz (Hz) in this order. Mass spectrometry (MS) was conducted at 70 eV using a Finnigan SSQ 7000 spectrometer (Thermo Electron Corporation, Madison, WI, USA). Elemental analyses were performed using a Perkin-Elmer 2400 analyzer, and the results showed agreement (±0.30) with the calculated values. To verify the purity of chemicals and monitor reactions, thin-layer chromatography was conducted on aluminum sheets precoated with silica gel (type 60 F_254_, Merck, Darmstadt, Germany). Solvents and chemical reagents were supplied by Sigma-Aldrich (Munich, Germany).

To analyze the morphology of the nanocomposites produced, small droplets of the liquid were placed on carbon-coated grids for TEM examination. The TEM images were acquired utilizing the TEM model JEOL 2010 FasTEM & Digital Micrograph User’s Guide (JEOL, Tokyo, Japan) at the Nanotechnology Center of Cairo University in Giza, Egypt. Scanning electron microscopy (SEM) images were taken with a JEOL JSM7600F instrument (JEOL, Tokyo, Japan) operating at 20 kV, and the measurements were also carried out at the Nanotechnology Center of Cairo University in Giza, Egypt. The composition analysis of the fabricated nanocomposites was conducted using energy-dispersive X-ray (XRD) spectroscopy. XRD data were collected using an XRD D8 Discovery-Bruker Company device (Coventry, UK) at 40 kV and 40 AM (1600 W) with a scan speed of 0.01 and a 2(θ) range of 10° to 80°. All the measurements were performed at the Nanotechnology Center of Cairo University in Giza, Egypt.

### 2.3. Animals and Experimental Study

Male Wistar rats weighing approximately 161 ± 11 g were utilized in this investigation. They were provided with a pellet diet and had access to water while being housed under controlled environmental conditions. The experimental procedures involving the rats adhered to the guidelines set forth by the International Care and Use of Laboratory Animals (Code: 86/609/EEC). Gastric ulcers were induced by administering 5 mL/kg of EtOH to the rats [32]. A total of 65 rats were used, consisting of 60 rats with gastric ulcers and 5 normal rats. These rats were divided into thirteen groups. The first group comprised normal rats (labeled as Con), while the second group consisted of rats with ulcers (labeled as GU). The remaining eleven groups included rats with gastric ulcers treated with ST, three Py-ST-X derivatives, three Py-ST-X derivative silver nanoparticles, Py-ST-X derivative tubes, and omeprazole (OPZ) at a single dose of 20 mg/kg body weight dissolved in distilled water. The total area of ulcers was determined using an inverted microscope equipped with a digital camera and analyzed with Image J software (Version 1.54i), along with digital measurements provided by an e-ruler. The content of gastric mucosa was determined following the method described by Ofusori et al. [40]. The ulcer index (UI) was calculated using the formula Ulcer index = (Ulcer area/Total mucosa surface area) × 100. Additionally, the curative index was determined using the formula Curative ratio = (US control − US treated)/US control [16]. The quantity of protein was determined using the Biuret colorimetric method (Biolabo Kit, Maizy, France, ref K2016). Myeloperoxidase (MPO) activity in gastric juice was measured spectrophotometrically at 460 nm, with one unit of MPO activity defined as the degradation of one μmol of peroxide per minute at 25 °C [41]. The assessment of gastric mucosa H^+^/K^+^ATPase activity followed the protocol outlined by Nagaya et al. [42]. The enzyme solution was combined with 10 mM KCl in a total volume of 1 mL of the assay medium, comprising 70 mM Tris buffer at pH 6.8 and 5 mM MgCl_2_ and then incubated for 1 h. Subsequently, 2 mM ATP was introduced to initiate the reaction, which proceeded for 20 min at 37 °C before being terminated by 10% trichloroacetic acid (TCA). Following centrifugation, the supernatant was mixed with 2.5 mL of ammonium molybdate and 0.5 mL of l-amino-2-naphthol-4-sulphonic acid, and the absorbance was measured at 620 nm. Results were expressed as mmol of Pi liberated per minute per milligram of protein. Gastric juice was collected by centrifuging the gastric mucus at 3500× *g* for 5 min at 4 °C to remove insoluble mucosal gastric. Stomach mucosal TBARS rates were calculated using the method outlined by Buege and Aust [43]. Gastric juice H_2_O_2_ concentrations were assessed using the protocol outlined by Dingeon et al. [44] with results presented as micromoles of H_2_O_2_per milligram of protein. 

### 2.4. Evaluation of Toxicity of Compounds on Rats

Bioactive derivatives with bromine (Br) or nitro (NO_2_) combined with carbon nanotubes were administered by gavage at dose 100 mg/kg daily to male rats. The rats were monitored every 4 h for 7 days post-administration. The serum glucose level was calculated spectrophotometrically at 505 nm (Kit Biolabo France, ref 26019). The serum total cholesterol (TC), low-density lipoprotein cholesterol (LDL-C), GOT, and GOT and GPT activities, as well as urea and creatinine levels, were quantified using commercial kits obtained from Biomaghreb (La Goulette, Tunisia). The kits used were Biomaghreb ref. 11022 and 10025 for GOT and GPT, and ref. 30023 and 25029 for urea and creatinine, respectively, which were calculated using commercial kits (Kits, Biolabo, France, ref 80106, 80019, and 90206). For histological analysis, fragments of gastric tissues were fixed in 10% formaldehyde and embedded in paraffin wax. These samples were then sectioned into 5 µm-thick histological sections and stained with hematoxylin and eosin (H&E) solution. The stained sections were examined and photographed using an Olympus CX41 light microscope (Olympus, Tokyo, Japan).

### 2.5. Evaluation of Antibacterial Activity

The susceptibility tests were conducted following the guidelines set by the National Committee for Clinical Laboratory Standards (NCCLS) in 1993. Screening experiments regarding the inhibitory zone were carried out for the newly synthesized compounds using the well diffusion approach [45]. Mueller–Hinton broth was inoculated with the inoculum suspension prepared from colonies grown overnight on agar plates (fungi were cultivated using malt broth). Mueller–Hinton agar plates were inoculated using a sterile brush dipped in the solution (fungi were cultivated on malt agar plates, and bacteria were cultivated on nutrient agar plates). To determine the minimum inhibitory concentration (MIC) value, the chemicals were dissolved in varying concentrations of dimethyl sulfoxide (DMSO). After 48 h at 28 °C for fungi and 24 h at 37 °C for bacteria, the inhibitory zone surrounding each well was measured [45]. The full list of analyzed microorganisms included two clinical fungi *Aspergillus fumigatus* (RCMB 002008) and *Candida albicans* (RCMB 005003 (1) ATCC 10231)), one Gram-positive bacterium (*Staphylococcus aureus* ATCC 25923), and one Gram-negative bacterium (*Escherichia coli* ATCC 25922).

### 2.6. Statistical Analysis

The data from each analysis were inputted into StatView 5.0 for statistical analysis. Results are expressed as means ± standard deviation (SD). Differences were assessed using the Fisher test. Statistical significance between groups was considered at *p* ≤ 0.05.

## 3. Results and Discussion

### 3.1. Preparation and Characterization of **Py-ST-X**

The **Py-ST-X** derivatives were efficiently synthesized through multicomponent reaction of compound **ST**, malononitrile, and various para-substituted benzaldehydes in the presence of ammonium acetate (Scheme 1). The spectroscopic data of synthetized heterocycles are in accordance with related compounds [46,47]. The IR spectra displayed characteristic absorption bands for new C≡N and NH_2_ groups. The ^1^H NMR spectra of **Py-ST-X** revealed the appearance of a new signal for the NH_2_ group exchangeable with D_2_O in addition to the disappearance of the signal corresponding to active thiazolomethylene protons. Furthermore, ^13^C NMR spectra show a distinct signal corresponding to the C≡N group but no signal for the C=O group. 

### 3.2. Morphology and Structure of AgNPs and CNTs

**Figure 1:**
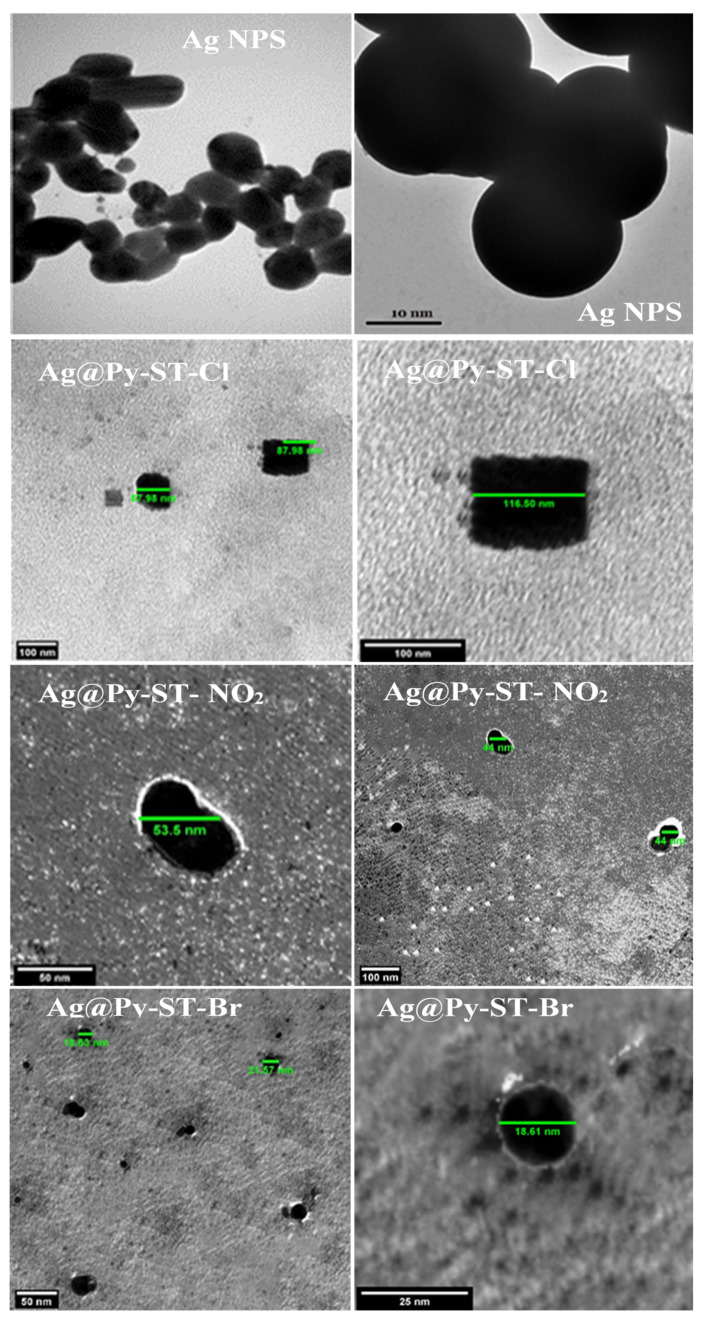
TEM images of AgNPs and **Ag@Py-ST-X** composites.

The first technique employed to determine the nanostructure of the **Py-ST-X** compounds with Ag nanoparticles is TEM. The TEM micrographs presented in Figure 1 depict the AgNPs alone and in the presence of the **Py-ST-X** heterocycles. Ag NPs appear spherical with a polycrystalline structure with an average size of 20 nm. Upon capping by **Py-ST-X,** the latter molecules formed nanoshells around Ag NPs, thus preventing their aggregation. 

In fact, the presence of NH_2_ group in **Py-ST-X** heterocycles facilitates the assembly of these molecules on Ag NPs by chemosorption [48] which reduces the hybrid components’ surface charge and enhances their stability, resulting in a reduced overall tendency to aggregate. 

Metal particles in colloidal solutions often carry a negative charge due to adsorbed anions, leading to increased aggregation [39]. Previous studies have demonstrated that combining nanoparticles with neutralized molecules, such as **Py-ST-X** molecules, can mitigate this aggregation tendency. These molecules neutralize adsorbed ions, reducing the particle surface charge and thereby inhibiting aggregation [39]. 

**Figure 2:**
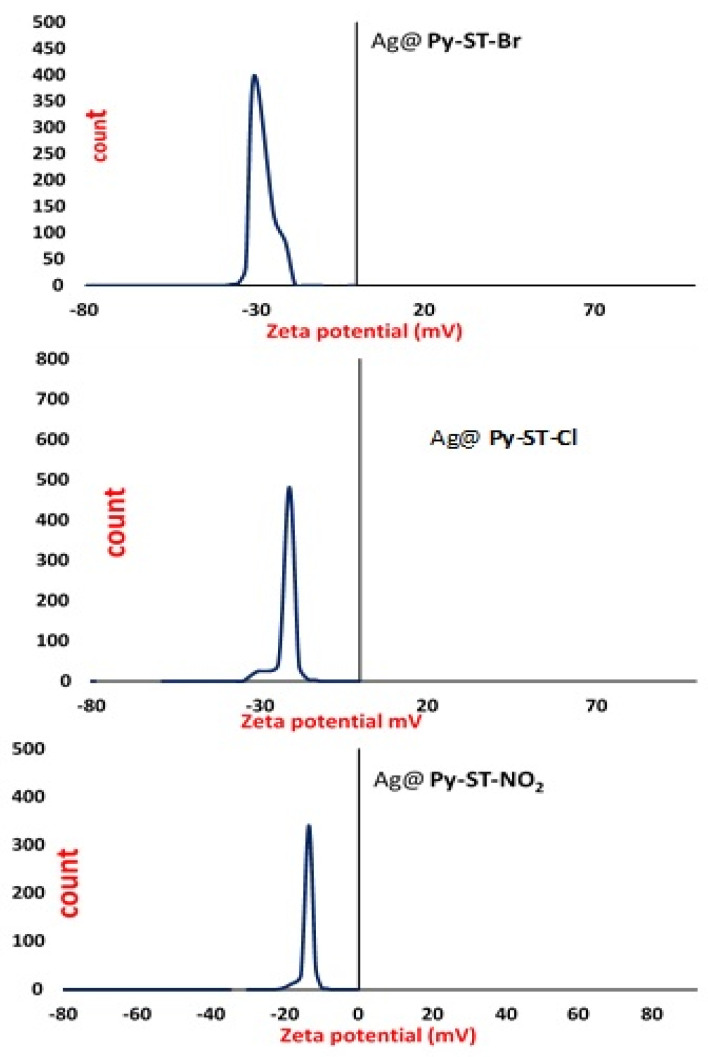
Zeta potential spectra of **Ag@ Py-ST-X** composites.

Zeta potential (ζ) measures the stability of the colloidal solution and the surface charge. The values deduced from the (ζ) spectra (Figure 2), −30 mV, −24 mV, and −13 mV for X = Br, Cl, and NO_2_ respectively reveals that the nanocomposites are more stable from electrostatic considerations with decreasing surface charge and aggregation tendency. 

**Figure 3:**
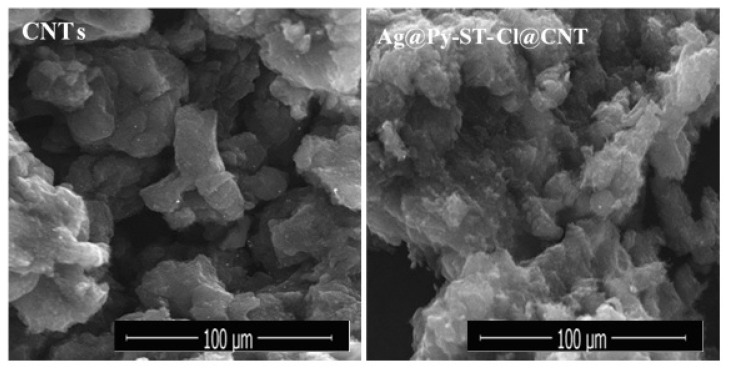
The SEM image of CNTs and Ag@Py-ST-Cl@CNT.

In this investigation, we have utilized SEM technique to examine the surface morphology of both CNTs and the synthetized **Ag@Py-ST-Cl@CNT** nanocomposite. Figure 3 illustrates SEM images of CNTs, showing the wide and smooth spaces. In Figure 3, CNTs image showed the wide and smooth spaces between their layers. Upon introducing Ag NPs coated with **Py-ST-Cl** molecules, these inter-layer spaces appear are filled, indicating that the Ag NPs coated with **Py-ST-Cl** molecules in the synthesized **Ag@Py-ST-Cl** nanocomposite effectively occupied these gaps [48].

**Figure 4:**
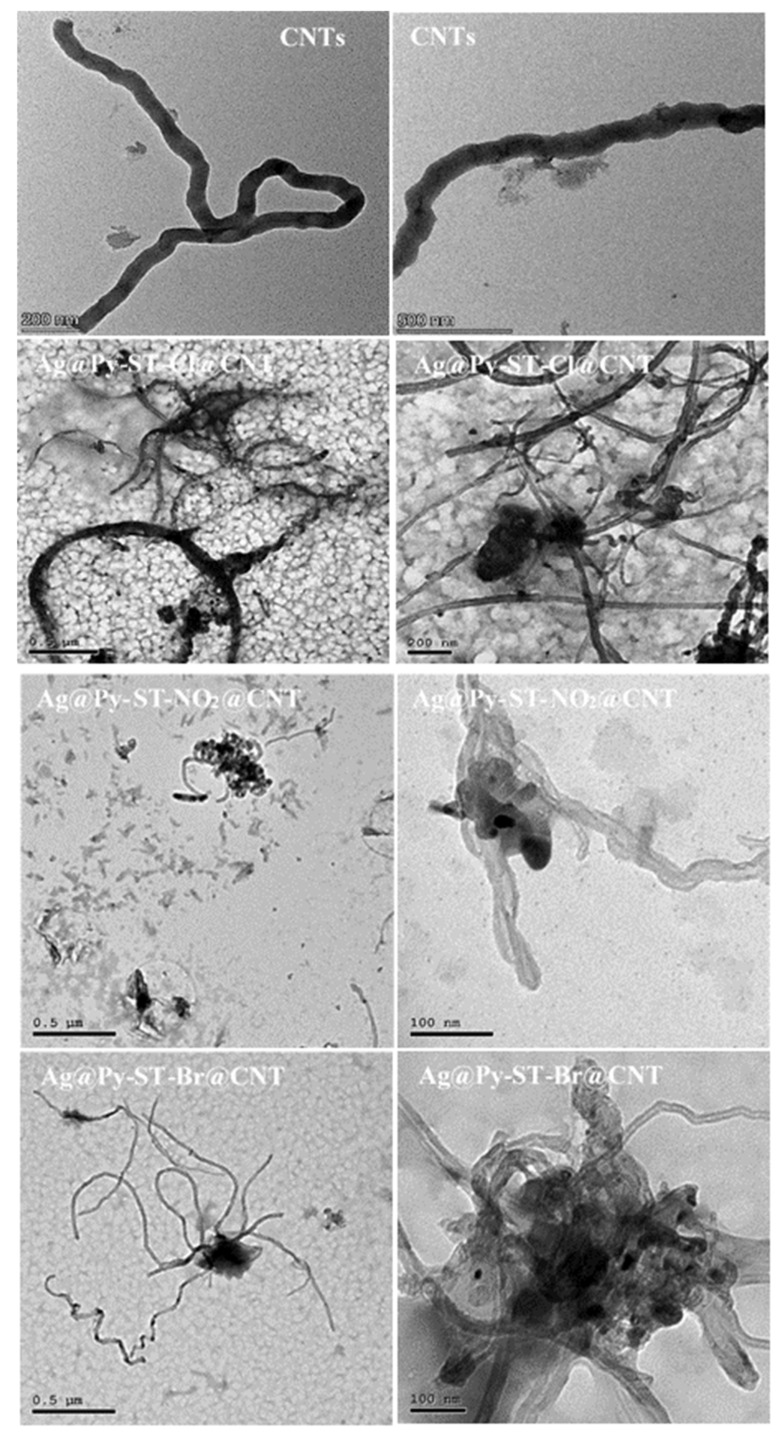
TEM image of CNTs and Ag@Py-ST-X@CNT.

**Figure 5:**
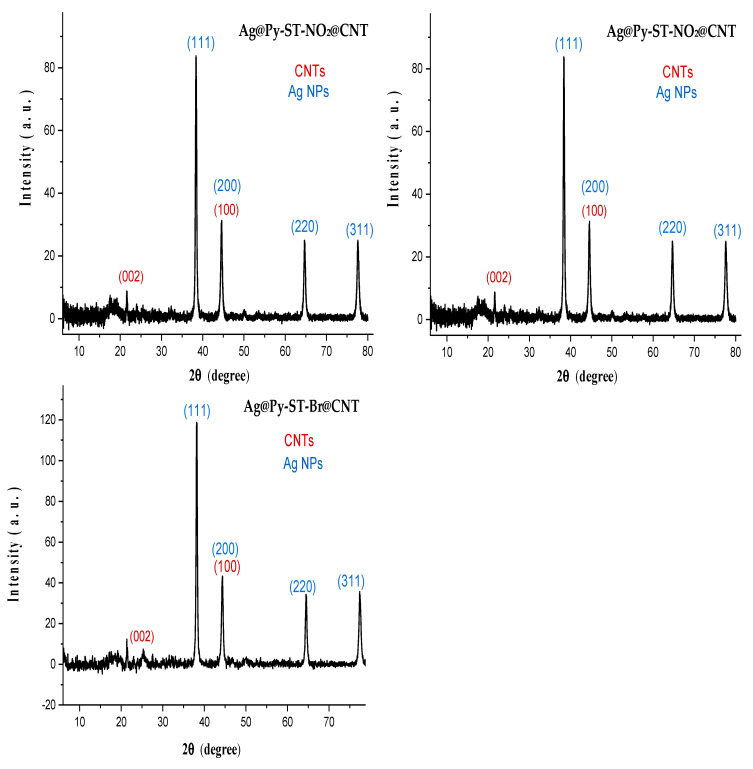
The XRD image of Ag@Py-ST-X@CNT.

The CNTs and the resulting nanocomposite **Ag@Py-ST-X@CNT** were further investigated using TEM (Figure 4). As depicted in Figure 3 and Figure 4, the silver nanoparticles have adhered firmly to the CNTs’ surface without aggregation. The morphology of the prepared nanocomposites **Ag@Py-ST-X@CNT**, (X = NO_2_, Cl, Br) underwent further examination using XRD, as depicted in Figure 5. 

The diffractograms reveal diffraction peaks assigned to the CNTs and silver (Ag) cristallographic planes, which affirms the successful synthesis of these nanocomposites. The significantly weakened peaks at 21.6° and at 44.5° are attributed respectively to the (002) and (100) planes of the carbon atoms hexagonal structure (JCPDS card No. 025-0284). Their low intensity arises from the smaller size tubular graphitic particles after interpenetration with **Py-ST-X**-coated SNPs molecules [49,50,51]. Furthermore, the important feature is that the XRD patterns of the composites reveal new peaks at 38.4°, 44.5°, 64.7°, and 77.6°, assigned to silver (Ag) nanoparticles crystallographic planes (111), (200), (220), and (311) respectively (JCPDS Card No. 001–1167) [50].

The average crystal size of Ag nanoparticles (diameter *D*) can be estimated by Scherrer’s formula expressed by Equation (1):

(1) $$D=\frac{K\lambda }{\beta \cos\theta }$$

where *λ* = 1.54 Å is the wavelength of X-rays, *K* = 0.9 is the empirical Scherrer constant, *θ* is the diffraction peak angular position, and *β* is its full width at half-maximum (FWHM). 

Relative to the maximum peak at 2*θ* = 38.4°, assigned to the (111) plane, the average crystal size of Ag nanoparticles was estimated to be 23.5 nm, very close to that determined from TEM micrographs (nearly 20 nm). These results serve as evidence that the Ag nanoparticles coated with **Py-ST-X** molecules interpenetrate the CNT layers, hence confirming the formation of the hybrid **Ag@Py-ST-X@CNT** nanomaterials.

### 3.3. Biological Activities

#### 3.3.1. Evaluation of the Toxicity of Bioactive Derivatives with Bromine (Br) or Nitro (NO_2_) with Carbon Nanotubes

Our results show that the administration of two bioactive derivatives with bromine (Br) or nitro (NO_2_) combined with carbon nanotubes at a high dose (100 mg/kg), compared to the active dose tested (20 mg/kg), does not cause any change in liver toxicity indices (GOT and GPT activities), renal toxicity indices (creatinine and urea levels), or significant changes in metabolic indices (TC, LDL, and blood sugar levels) (Table 1). Additionally, no signs of physiological toxicity (mobility, behavior) were observed.

#### 3.3.2. Effect of New Derivatives of Spiro-Thiazolidine-Carbonitrile on Key Enzyme Related to Gastric Acidity and Ulceration as H^+^/K^+^ATPase

Gastrointestinal issues have emerged as a significant global concern in recent times. One prominent risk factor that has been implicated in causing damage to the gastric mucosa is ethanol. The consumption of alcohol is well known for its ability to induce stress ulcers (SU), also known as acute gastric mucosal lesions, which typically manifest as superficial injuries primarily in the fundus of the stomach. This induction is attributed to the heightened activity of H^+^, K^+^-ATPase, which leads to an increase in gastric acidity. Consequently, targeting H^+^/K^+^ ATPase has shown promise in the treatment of ulcers.

**Figure 6:**
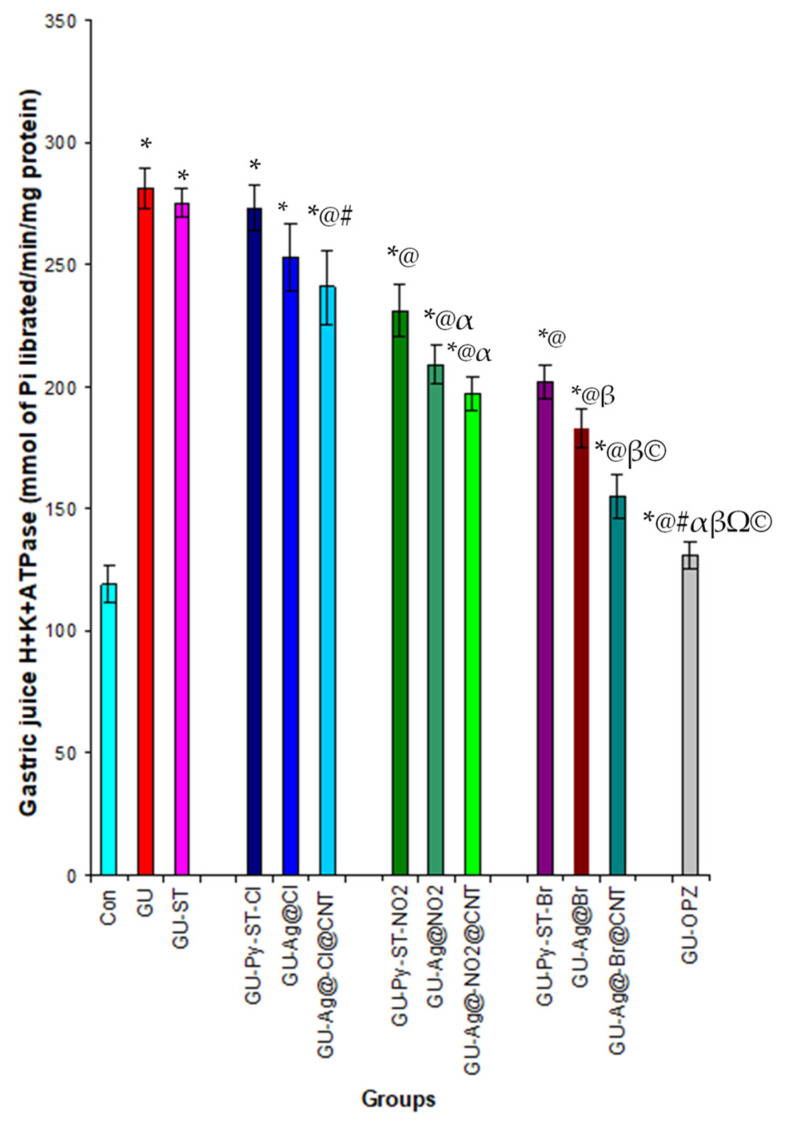
Impact of ingesting novel derivatives of spiro-thiazolidine-carbonitrile on H^+^/K^+^ATPase in gastric juice. Statistical analyses as given as * *p* < 0.05 significant differences compared to controls; @ *p* < 0.05 significant differences compared to GU rats; # *p* < 0.05 significant differences compared to GU rats treated with Py-ST-Cl. α *p* < 0.05 significant differences compared to GU rats treated with Py-ST-NO2. β *p* < 0.05 significant differences compared to GU rats treated with Py-ST-Br. Ω *p* < 0.05 significant differences compared to GU rats treated with Py-ST-Ag@NO_2_. © *p* < 0.05 significant differences compared to GU rats treated with Py-ST-Ag@Br.

The findings of our study indicate that the ingestion of ethanol triggers the activation of enzymes, particularly H^+^/K^+^ATPase activity, by 136% compared to normal rats. Treatment with ST and the **Py-ST-Cl** derivative results in a slight and statistically insignificant reduction. On the other hand, supplementation with **Py-ST-NO_2_** and **Py-ST-Br** derivatives leads to a decrease in H^+^/K^+^ATPase activity by approximately 9 and 14%, respectively, when compared to untreated rats with gastric ulcers. Furthermore, the incorporation of these two derivatives into silver nanoparticles enhances their inhibitory activity. Specifically, **Ag@Py-ST-NO_2_** and **Ag@Py-ST-Br** show a 10 and 13% increase respectively, compared to the derivatives without silver nanoparticles. Additionally, the nano-tubulation of **Ag@Py-ST-NO_2_** and **Ag@Py-ST-Br** potentially increases the inhibitory activity of the key gastric acidity enzyme, H^+^/K^+^ATPase, in gastric juice by 27 and 45%, respectively, when compared to untreated rats (Figure 6).

In fact, incorporating these two derivatives into SNPs enhanced their inhibitory activity against this enzyme compared to the derivatives without silver nanoparticles. As reported in previous studies [50,51,52,53,54,55], silver-based nanocomposites promotes the interaction of heterocyclic compounds with human tissues and their absorption and subsequently increases biological activities such as antidiabetic, anti-inflammatory, and antioxidant properties, which may improve the protection of gastric tissues. Additionally, the nano-tubulation of **Ag@Py-ST-NO_2_** and **Ag@Py-ST-Br** potentially enhances the absorption of the bioactive compound at both gastric and intestinal levels, thereby exerting more inhibitory effects against H^+^/K^+^ATPase or further down-regulating gastric acidity. Furthermore, the incorporation of our compounds into silver nanocomposites potentially neutralizes the free radicals released during ethanol metabolism, subsequently reducing damage to gastric tissues. Even more intriguingly, the nano-tubulation of **Ag@Py-ST-NO_2_** and **Ag@Py-ST-Br** potentially boosts the stability of the compounds and increases the absorption of the bioactive compound at both gastric and intestinal levels, thereby amplifying inhibitory effects against H^+^/K^+^ATPase or further regulating gastric acidity downward. Moreover, integrating our compounds into silver nanocomposites potentially counteracts the free radicals released during ethanol metabolism, subsequently mitigating damage to gastric tissues.

#### 3.3.3. Effect of New Derivatives of Spiro-Thiazolidine-Carbonitrile on Gastric Inflammation

**Figure 7:**
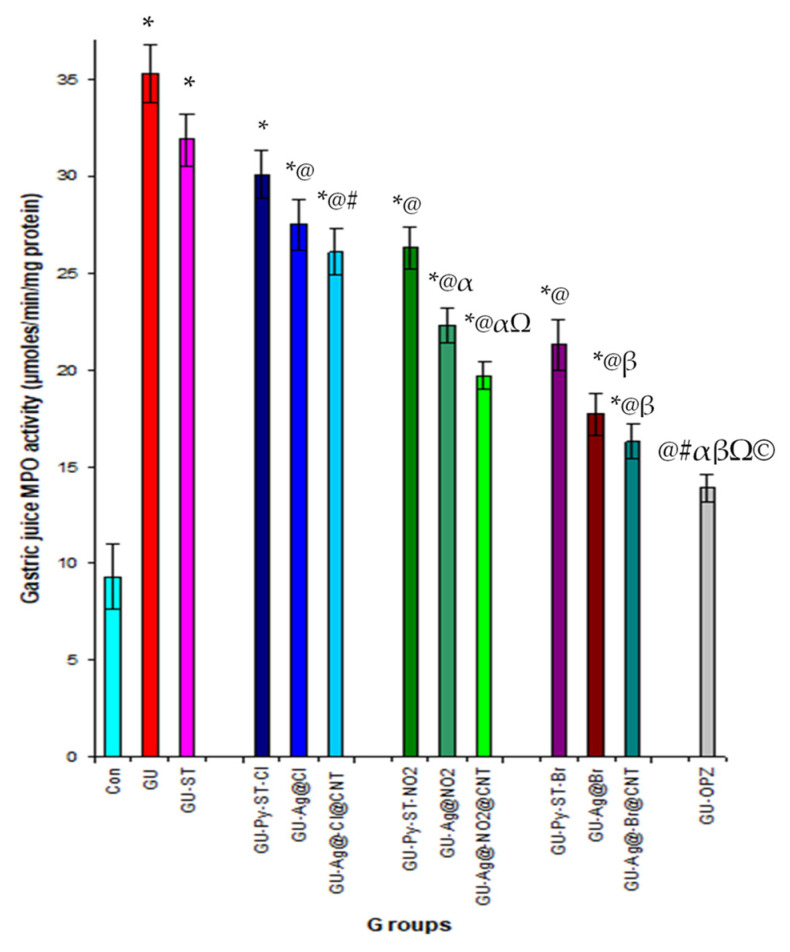
Impact of ingesting novel derivatives of spiro-thiazolidine-carbonitrile on neutrophil infiltration enzyme index and gastric inflammation. Statistical analyses as given as: * *p* < 0.05 significant differences compared to controls; @ *p* < 0.05 significant differences compared to GU rats; # *p* < 0.05 significant differences compared to GU rats treated with Py-ST-Cl. α *p* < 0.05 significant differences compared to GU rats treated with **Py-ST-NO_2_**. β *p* < 0.05 significant differences compared to GU rats treated with Py-ST-Br. Ω *p* < 0.05 significant differences compared to GU rats treated with **Py-ST-Ag@NO_2_**. © *p* < 0.05 significant differences compared to GU rats treated with **Py-ST-Ag@Br**.

The findings from Figure 7 show that ethanol consumption is linked to an increase in gastric inflammation, with a 279% rise in MPO enzyme activity indicating neutrophil infiltration in comparison to control rats. The use of ST does not affect gastric inflammation. However, our study suggests that the consumption of new spiro-thiazolidine-carbonitrile derivatives leads to a slight reduction in inflammation. Specifically, the use of derivatives like **Py-ST-Cl**, **Py-ST-NO_2_**, and **Py-ST-Br** resulted in a 22 and 26% decrease in MPO activity, respectively. Moreover, incorporating these derivatives into SNPs enhances their inhibitory effects, with **Ag@Py-ST-NO_2_** and Ag@Py-ST-Br showing a 15% and 18% increase, respectively, compared to derivatives without SNPs. Additionally, the nano-tubulation of **Ag@Py-ST-NO_2_** and Ag@Py-ST-Br potentially enhances the inhibitory activity of MPO enzyme, a crucial factor in gastric inflammation, by 49 and 54%, respectively, compared to untreated rats.

Based on our findings, the inclusion of these ST derivatives in silver nanoparticles has been shown to enhance their effectiveness in inhibiting gastric juice MPO. Furthermore, the nano-tubulation of **Ag@Py-ST-NO_2_** and **Ag@Py-ST-Br** has the potential to further amplify MPO inhibition, which is a crucial enzyme associated with gastric inflammation. This improvement can be attributed to the impact of silver nanocomposites, particularly carbon nanotubulation, in increasing the stability of our bioactive compounds within the gastric environment and facilitating their absorption. Previous research has highlighted the notable anti-inflammatory properties of thiazolidine derivatives, with Br or NO_2_ substitutions further enhancing this trait. Additionally, our results support the findings of Ebenezer et al. [56], suggesting that integrating thiazolidines into silver nanoparticles enhances their anti-inflammatory capabilities. This enhancement is attributed to two mechanisms: firstly, their role in enhancing gastric stability and absorption, and secondly, their interactions with tissues and cells. Holla et al. [57] have documented that substituting Br or NO_2_ in thiazole derivatives enhances and stimulates the anti-inflammatory activity of the compound. Indeed, the presence of bromine (Br) or nitro (NO_2_) substituents in compounds has been linked to increased anti-inflammatory activity, as observed in various studies.

#### 3.3.4. Effect of New Derivatives of Spiro-Thiazolidine-Carbonitrile Nanocomposites Stress Oxidant in Gastric Tissues

**Figure 8:**
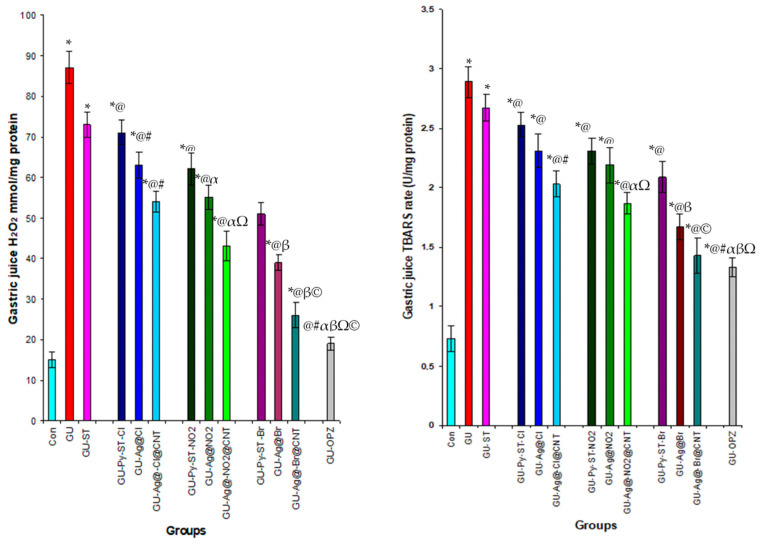
Impact of ingesting new spiro-thiazolidine-carbonitrile derivatives on oxidative stress indices such as H_2_O_2_ and TBARS in rats. Statistical analyses as given as: * *p* < 0.05 significant differences compared to controls; @ *p* < 0.05 significant differences compared to GU rats; # *p* < 0.05 significant differences compared to GU rats treated with **Py-ST-Cl**. α *p* < 0.05 significant differences compared to GU rats treated with **Py-ST-NO_2_**. β *p* < 0.05 significant differences compared to GU rats treated with Py-ST-Br. Ω *p* < 0.05 significant differences compared to GU rats treated with **Py-ST-Ag@NO_2_**. © *p* < 0.05 significant differences compared to GU rats treated with Py-ST-Ag@Br.

The results of this investigation demonstrate that the presence of gastric tissue ulceration is linked to a significant rise in oxidative stress indicators, such as the concentrations of H_2_O_2_ and TBARS in gastric fluid (Figure 8). The administration of ST does not have any effect on these indicators. However, the introduction of ST derivatives leads to a notable decrease in the levels of H_2_O_2_ and TBARS, with the most substantial reduction observed in rats treated with **Py-ST-Br**, resulting in reductions of 37% and 29%, respectively. Furthermore, the incorporation of these derivatives into silver nanoparticles significantly boosts their capacity to counteract free radicals such as H_2_O_2_. Interestingly, the nanostructuring of **Ag@Py-ST-Cl**, **Ag@Py-ST-NO_2_**, and **Ag@Py-ST-Br** nanoparticles seems to enhance their ability to neutralize harmful free radicals.

Our results indicate that the introduction of bromine (Br) or nitro (NO_2_) substitutions in spiro-thiazolidine compounds results in significant yet moderate antioxidant properties in gastric tissues when compared to untreated ulcers [58,59,60]. Furthermore, our research illustrates that the combination of spiro-thiazolidine with Br or NO_2_ in silver nanocomposites substantially boosts this antioxidant activity by decreasing the levels of H_2_O_2_ and TBARS in gastric juice [59]. The most powerful antioxidant effect was observed in ulcerated rats treated with spiro-thiazolidine with Br or NO_2_ incorporated into carbon nanotubes, underscoring the importance of pharmaceutical techniques in enhancing the stability and bioavailability of bioactive compounds in gastrointestinal tissues. These findings align with previous studies suggesting that Br or NO_2_ substitution in spiro-thiazolidine derivatives amplifies the antioxidant capacity of the compound [60]. In addition to their diverse applications in various fields, numerous prior investigations have focused on exploring the antioxidant properties of silver nanoparticles [61]. This is due to their ability to counteract free radicals and their role in improving the stability of different bioactive compounds. Ahn et al. [62] examined the antioxidant potential of thirty plant extracts and the silver nanoparticles (AgNPs) produced from these extracts. Their study concluded that the scavenging activity, as evaluated by the DPPH assay, increased with higher concentrations of either the plant extract or the AgNPs.

#### 3.3.5. Effect of New Derivatives of Spiro-Thiazolidine-Carbonitrile Nanocomposites on Gastric Tissues Ulceration

**Figure 9:**
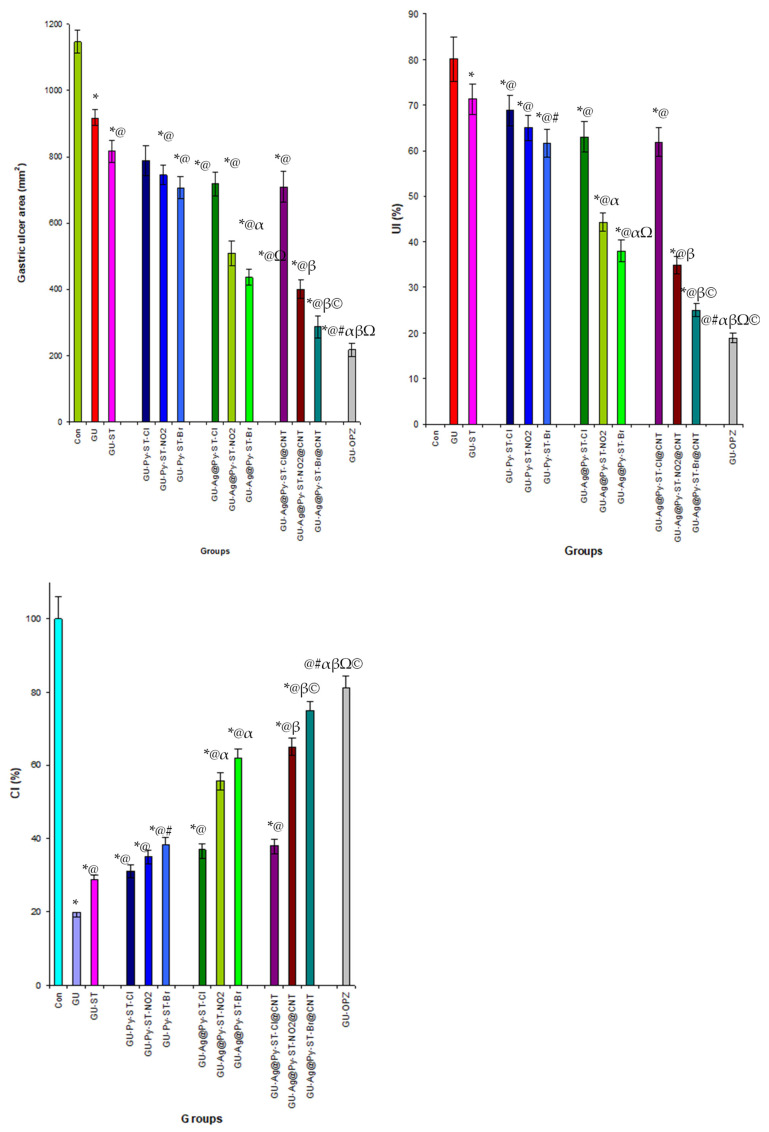
Effect of new spiro-thiazolidine-carbonitrile derivatives on gastric ulcer surface, ulcer index, and curative index. Statistical analyses as given as: * *p* < 0.05 significant differences compared to controls; @ *p* < 0.05 significant differences compared to GU rats; # *p* < 0.05 significant differences compared to GU rats treated with **Py-ST-Cl**. α *p* < 0.05 significant differences compared to GU rats treated with **Py-ST-NO_2_**. β *p* < 0.05 significant differences compared to GU rats treated with Py-ST-Br. Ω *p* < 0.05 significant differences compared to GU rats treated with **Py-ST-Ag@NO_2_**. © *p* < 0.05 significant differences compared to GU rats treated with Py-ST-Ag@Br. Our findings clearly indicate that derivatives with chlorine (Cl) exhibit no discernible effect in various forms. Conversely, derivatives with bromine (Br) or nitro (NO_2_) display gastric anti-ulcer activities. Furthermore, incorporating these derivatives into silver nanoparticles enhances their inhibitory effects. Additionally, the tubulation of these compounds enhances their biological interaction and anti-ulcer activity.

The obtained results (Figure 9) indicate that ethanol ingestion is correlated with an 80% increase in the surface area of gastric tissue ulceration [63,64]. Administration of **ST** in rats with gastric ulcers results in a slight reduction in ulcer index (UI), decreasing it to 71%. Supplementation with ST derivatives decreases the rate of ulceration, with the most pronounced effect observed in ulcerated rats treated with **Py-ST-Br**, achieving a UI of 61%.

Furthermore, incorporating these derivatives into silver nanoparticles significantly enhances their ability to protect gastric tissues from ulceration. **Ag@Py-ST-Cl**, **Ag@Py-ST-NO_2_**, and **Ag@Py-ST-Br** demonstrate notable immunization of the surface of ulcerated gastric tissues, with reduction rates in ulcer index of 63%, 44%, and 38%, respectively. Moreover, nano-tubulation of **Ag@Py-ST-NO_2_** and **Ag@Py-ST-Br** potentially amplifies the protective ability of these compounds against gastric tissue damage and ulceration. This is evidenced by a significant reduction in the rate of ulceration of gastric tissues by 21, 56, and 68%, respectively compared to untreated ulcers. The enhancement of anti-gastric ulcer (GU) efficacy resulting from the incorporation of Br and NO_2_ heterocyclic compounds into silver nanocomposites, specifically within carbon nano-tubes, can be attributed to the potent anti-inflammatory and antioxidant properties of these nanotubes, along with the improved stability and bioavailability of the bioactive compounds. Teixeira et al. [63] found that the administration of thiazolidinone derivatives protects gastric tissues from ulceration by reducing levels of inflammatory cytokines and modulating the oxidative pathway. Additionally, we observed either minimal inhibition or a lack of activity for the other compounds tested.

#### 3.3.6. Effect of New Derivatives of Spiro-Thiazolidine-Carbonitrile Nanocomposites on Histological Changes in Gastric Tissue Architecture

**Figure 10:**
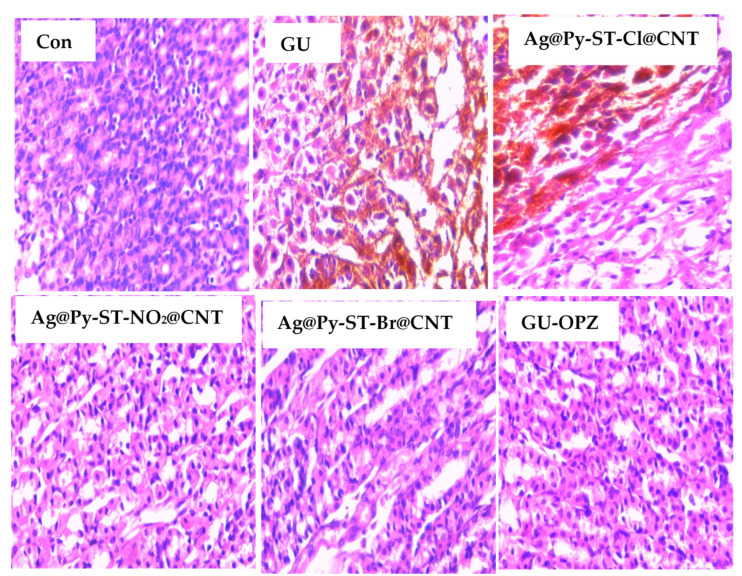
Impact of the administration of new spiro-thiazolidine-carbonitrile derivatives on the integrity of gastric tissues. Our results show that the ingestion of ethanol causes severe inflammation in gastric tissues, observed by a large amount of hemosiderin and siderophages. In rats treated with **Py-ST-Ag@NO_2_@CNT** and **Ag@Py-ST-Br@CNT**, a powerful anti-inflammatory activity was demonstrated by an absence of hemosiderin deposition and a strong suppression of siderophages, especially in rats treated with Br nanotubes (×400 magnification).

Our histological study, presented in Figure 10, reveals the normal histology of gastric tissue, showing a normal histological structure of the gastric mucosa and submucosa in the normal rat group (Con). Compared to the control group, the gastric architecture of untreated ulcerated rats presented serious histopathological alterations such as severe infiltration of lymphocytes, formation of siderophages, and severe deposition of hemosiderin in gastric tissues. 

In rats with gastric ulceration, no protective effect was observed, and the tissues appeared similar to those of untreated ulcerated rats. However, in ulcerated rats treated with **Py-ST-Ag@NO_2_@CNT** and **Ag@Py-ST-Br@CNT**, a potential protective effect on gastric tissues was demonstrated. This was evidenced by a reduction in siderophages and severe hemosiderin deposition in the gastric tissues. In fact, neutrophil influx is observed in various models of gastric ulcers, where it is considered a contributor to the damaging process. Studies [65,66] have shown that neutrophils quickly accumulate in tissue injured by acute Et/HCl-induced gastric lesions in mice [65,67]. Moreover, in vivo neutrophil depletion significantly reduced the injured area. These findings suggest that targeting neutrophil recruitment could be a viable strategy for anti-gastric ulcer therapy, and this process can be modulated by LYSO-7 treatment. Cerqua et al. [66] reported that spiro-thiazolidine potentially inhibits the activity of key inflammation-related enzymes such as cyclooxygenase (COX), 5-lipoxygenase (5-LOX), and 5-LOX-activating protein (FLAP). Additionally, it suppresses the infiltration of neutrophils, thereby protecting tissues from damage.

#### 3.3.7. Assessment of the Antifungal Efficacy of Novel Spiro-Thiazolidine-Carbonitrile Derivatives

**Figure 11:**
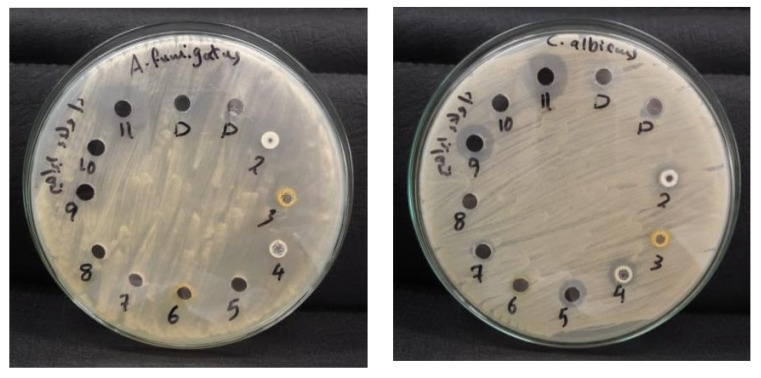
Antifungal activity of heterocycle derivatives via determination of the inhibition zone (mm) around discs impregnated with ST (2), Py-ST-Cl (3), Py-ST-NO_2_ (4), Py-ST-Br (5), Ag@Py-ST-Cl (6), Ag@Py-ST-NO_2_ (7), Ag@Py-ST-Br (8), Ag@Py-ST-Cl@CNT (9), Ag@Py-ST-NO_2_@CNT (10), Ag@Py-ST-Br@CNT (11), Ketoconazole (P), Dimethylsulfoxide (D).

The efficacy of the new compounds against two highly pathogenic fungi for humans, *Aspergillus fumigatus* and *Candida albicans*, was assessed. The results are summarized in Table 2 and the antifungal potency of heterocycles derivatives was qualitatively assessed by the presence or the absence of an inhibition zone diameter (Figure 11). 

The **Ag@Py-ST-Br@CNT** nanotubes exhibit the highest activity against these two fungi, with inhibition diameters of 22 mm and 18 mm, respectively. This inhibitory capacity exceeds that of the standard compound ketoconazole, which shows inhibition diameters against *Aspergillus fumigatus* and *Candida albicans* of 17 mm and 20 mm, respectively. Additionally, we observed limited inhibition or lack of activity for the other compounds tested. Nevertheless, the nanocomposite **Ag@Py-ST-X@CNT** displayed superior performance against the *Aspergillus fumigatus* (RCMB 002008) strain compared to the commonly used reference drug, and its efficacity was only moderate against all other strains when compared to the respective reference drugs for each strain. Furthermore, all strains exhibited a significant response to the nanocomposite **Ag@Py-ST-Cl@CNT**, except for *Aspergillus fumigatus* (RCMB 002008), which showed no response at all. Conversely, **Ag@Py-ST-X** showed some activity against all strains compared to the reference drug, although it had no effect on *Aspergillus fumigatus* (RCMB 002008). This clearly indicates that the incorporation of **Ag@Py-ST-Br** into CNTs enhances the interaction or penetration of compounds into fungal cells, leading to their destruction. In contrast, for other derivatives containing Cl or NO_2_, there is a lack of antifungal activity, possibly due to the absence of interaction between the derivatives and the fungal cells

#### 3.3.8. Assessment of the Antibacterial Efficacy of Novel Spiro-Thiazolidine-Carbonitrile Derivatives

**Figure 12:**
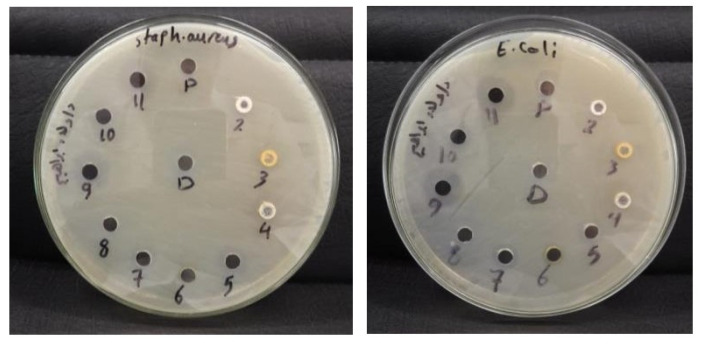
Antibacterial activity of heterocycle derivatives via determination of the inhibition zone (mm) around discs impregnated with ST (2), Py-ST-Cl (3), Py-ST-NO_2_ (4), Py-ST-Br (5), Ag@Py-ST-Cl (6), Ag@Py-ST-NO_2_ (7), Ag@Py-ST-Br (8), Ag@Py-ST-Cl@CNT (9), Ag@Py-ST-NO_2_@CNT (10), Ag@Py-ST-Br@CNT (11), Gentamycin (P), Dimethylsulfoxide (D).

The results depicted in Table 3 and Figure 12 showed that **Py-ST-X** derivatives exhibit weak inhibitory effects against pathogenic bacteria such as *Staphylococcus aureus* and *Escherichia coli*. Nevertheless, it was noted that the inclusion of these derivatives into SNPs significantly enhances their capacity to hinder the growth of these bacterial strains. Moreover, the nano-tubulation of **Ag@Py-ST-Cl** and **Ag@Py-ST-Br** further amplifies their inhibitory efficacy against microbes. However, in the case of **Ag@Py-ST-Br**, there is a complete absence of such activity. Among these, only the nanocomposite **Ag@Py-ST-NO_2_** exhibited minimal activity against the Gram-negative bacterium *Escherichia coli* (ATCC 25922), while **Ag@Py-ST-Cl** displayed minimal activity against both the Gram-negative bacterium *Escherichia coli* (ATCC 25922) and the Gram-positive *bacterium Staphylococcus aureus* (ATCC 25923). Additionally, the **Py-ST-NO_2_** compound showed no activity against Gram-positive bacteria (*Staphylococcus aureus* ATCC 25923) and fungi, but exhibited minimal activity against Gram-negative bacteria. Conversely, the **Py-ST-Br** compound demonstrated no effect against *Aspergillus fumigatus* (RCMB 002008) and displayed limited efficacy against Gram-positive bacteria, with no effect against Gram-negative bacteria. 

#### 3.3.9. Structure–Activity Relationship 

The primary objective of these studies was to examine the structure–activity relationships (SAR) of our Py-ST-X derivatives, specifically **Ag@Py-ST-X** and **Ag@Py-ST-Br@CNT**. Our findings indicate that **ST** derivatives containing chlorine (Cl) did not exhibit any effect, whereas those containing nitro (NO_2_) and bromine (Br) displayed weak activities against key enzymes involved in gastric acidity and inflammation. The inclusion of these derivatives into SNPs enhances and amplifies this activity. This suggests that the presence of both silver (Ag) and bromine (Br) in the same compound facilitates interaction with digestive tissue enzymes, such as H^+^/K^+^ATPase and MPO, resulting in a reduction in their genetic expression or activity. Furthermore, the nanotubulation of **Ag@Py-ST-NO_2_** and particularly **Ag@Py-ST-Br** promotes strong interaction with gastric tissues, offering protection against gastric emptying. Consequently, this leads to more enduring and potent effects against gastric acidity and inflammation.

Regarding the initial screening of the antimicrobial outcomes, it is evident that the presence of a pyridine ring attached to the spirothiazolidine skeleton enhances the antibacterial efficacy of the evaluated derivatives against the tested microorganisms [32]. Substitutions in the pyridine core at positions 3 and 2 play a crucial role in enhancing activity, with a highly nucleophilic group (NH_2_) at position 2 and an electron-withdrawing group at position 3 being preferred [32]. Conversely, antibacterial activity is heightened in derivatives where halogens such as chlorine and bromine are incorporated into the spirothiazolidine carbonitrile framework. Compounds containing chlorine atoms, such as Py-ST-Cl, **Ag@Py-ST-Cl**, and **Ag@Py-ST-Br@CNT**, demonstrated activity ranging from high to moderate against most pathogenic microbes. Additionally, the utilization of compounds that consist of halogen atoms along with carbon nano-tubes in the creation of nanocomposites seems to improve the biological effectiveness against the targeted pathogenic microorganisms. On the contrary, compounds that were prepared without halogen atoms exhibited minimal to no impact on the microorganisms. These findings highlight the significance of integrating halogen atoms alongside carbon nanotubes to boost the biological activity against pathogenic microorganisms.

## 4. Conclusions

Spiro-thiazolidine-carbonitrile compounds containing Br or NO_2_ groups have been found to possess anti-inflammatory, antioxidant, and significant anti-enzyme activities related to gastric acidity regulation and ulceration. These compounds act as relatively weak inhibitors of H^+^/K^+^ATPase. When these compounds are integrated into silver nanoparticles and carbon nanotubes, the therapeutic effects are enhanced, likely due to improved stability, bioavailability, absorption, and interaction with cells and tissues. Moreover, the use of carbon nanotubes, especially those containing bromine, has shown effectiveness against toxic microbes by aiding in their interaction and penetration into these cells. Therefore, the incorporation of activated compounds into silver nanoparticles and carbon nanotubes holds promise as a future technology for improving stability, interaction, absorption, penetration into target cells, and ultimately, the efficacy of treating various pathologies. 

## Figures and Tables

**Table 1:** The effect of **Ag@Py-ST-NO_2_@CNT** and **Ag@Py-ST-Br@CNT** at a dose of 100 mg/kg on TC, LDL-C, creatinine, urea, and blood glucose levels, as well as GOT and GPT activities in the serum of rats, was evaluated. No significant increase in these parameters was observed, indicating that these nanocomposites are non-toxic even at high doses (5 times the active dose).

	Con	Ag@Py-ST-NO_2_@CNT	Ag@Py-ST-Br@CNT
TC (g/L)	0.96 ± 0.09	1.01 ± 0.13	0.98 ± 0.08
LDL-C (g/L)	0.46 ± 0.02	0.51 ± 0.08	0.48 ± 0.11
GOT	31 ± 3.4	34 ± 2.5	33 ± 1.9
GPT	15 ± 1.7	18 ± 1.3	17 ± 2.1
Glycemia (g/L)	0.97 ± 0.13	0.99 ± 0.35	0.96 ± 0.51
Creat (mg/L)	11.3 ± 1.1	12.9 ± 1.8	12.1 ± 1.3
Urea (g/L)	0.75 ± 0.17	0.81 ± 0.1	0.79 ± 0.17

**Table 2:** Mean zone of inhibition in mm produced on a range of pathogenic microorganisms. Results are given in the following table: (Positive control for fungi Ketoconazole 100 μg/mL).

Sample Code Microorganisms	Py-ST-X			Ag@Py-ST-X			Ag@Py-ST-X@CNT			Ketoconazole
	X=Cl	X=NO_2_	X=Br	X=Cl	X=NO_2_	X=Br	X=Cl	X=NO_2_	X=Br	
*A. fumigatus * (RCMB 002008)	15	NA	NA	NA	NA	NA	NA	NA	22	17
*C. albicans* RCMB 005003	11	NA	12	12	NA	NA	16	NA	18	20

**Table 3:** Mean zone of inhibition in mm produced on a range of pathogenic microorganisms. Results are depicted in the following table: (Gentamycin: 4 µg/mL).

Microorganisms	Py-ST-X			Ag@Py-ST-X			Ag@Py-ST-X@CNT			Gentamycin
	X=Cl	X=NO_2_	X=Br	X=Cl	X=NO_2_	X=Br	X=Cl	X=NO_2_	X=Br	
*S. aureus* (ATCC 25923)	11	9	9	12	NA	NA	16	NA	15	24
*E. coli* ATCC 25922	NA	NA	NA	12	9	NA	17	NA	18	30

## Data Availability

The data is contained within the manuscript.
